# Insomnia and depression in Chinese academic researchers: mediation by anxiety and resilience with differences among researchers by educational level

**DOI:** 10.3389/fpsyt.2025.1709399

**Published:** 2025-11-21

**Authors:** Lin Han, Yuanrui He, Botao Gong, Tingfeng Liu, Puqiao Wen, Mingjun Zhong, Qinshuo Ren, Hui Wang, Xudong Chen

**Affiliations:** 1 Department of Psychiatry, National Clinical Research Center for Mental Disorders, and National Center for Mental Disorders, The Second Xiangya Hospital of Central South University, Changsha, Hunan, China; 2 The Second Affiliated Hospital, Hunan University of Chinese Medicine, Changsha, Hunan, China

**Keywords:** insomnia, depression, resilience, education level, researcher

## Abstract

**Introduction:**

Mental health issues such as insomnia may exhibit unique interaction patterns among high-stress populations, with educational attainment potentially playing a distinctive role. However, the current mental health status of researchers at the forefront of scientific advancement has not garnered widespread attention. This study tested whether anxiety and psychological resilience jointly mediate the association between insomnia and depressive symptoms among Chinese researchers, and whether these pathways are moderated by educational attainment.

**Methods:**

A cross-sectional online survey was conducted among researchers from 19 provinces in China between September and November 2023, and valid responses from 645 participants were analyzed using the Insomnia Severity Index, the Generalized Anxiety Disorder-7 as a measure of generalized anxiety symptoms, the Patient Health Questionnaire-9, and the Connor–Davidson Resilience Scale. Partial correlation and chain mediation analysis were applied to examine the relationships between insomnia, anxiety, resilience, and depression, while multi-group structural equation modeling (SEM) assessed differences between researchers with and without doctoral degrees.

**Results:**

The proportion meeting the ISI screening threshold (≥ 8) was higher in researchers aged ≥ 40 and in those with doctoral degrees. In mediation models, insomnia severity demonstrated a direct association with depressive symptoms (effect: 38.31%) and indirect associations via anxiety and resilience (total effect: 61.69%). Specifically, the chain mediation effect formed by anxiety and resilience showed a suppressing effect of 0.66%, while the independent mediating effects of anxiety and resilience were 64.36% (enhancing effect) and 2.01% (suppressing effect), respectively. Multi-group SEM analyses further revealed stronger anxiety-resilience-depression linkages in researchers without doctoral degrees, highlighting their heightened vulnerability.

**Discussion:**

Overall, insomnia is centrally associated with depression among researchers through a dual pathway involving anxiety and resilience, with the protective role of resilience being more complex among non-doctoral researchers, underscoring the need for targeted interventions such as sleep hygiene programs and resilience training to mitigate mental health risks in academic settings.

## Introduction

Scientific researchers constitute a professional group at heightened risk of psychological distress, with higher rates of insomnia, anxiety, and depression compared to the general workforce (1). Similar patterns have been observed in other cognitively intensive occupational groups such as female scientists (2) and healthcare workers (3), underscoring that such professionals face heightened vulnerability in sleep and mood disturbances. Although demanding workloads, competitive promotion requirements being widely recognized as external pressures, mental health levels are the central concern requiring attention, which remain insufficiently investigated in this population. Recent large-scale studies indicate that psychological morbidity among researchers is not only more prevalent than in many other professional groups, but also tends to be more persistent, underscoring the need for exploring its underlying mechanisms (4, 5).

Insomnia has been a salient risk factor in this context. Beyond its high prevalence in knowledge-intensive populations, insomnia is now established as a causal antecedent for depression (6). Longitudinal and meta-analytic evidence across diverse cohorts consistently shows that sleep disturbances predict subsequent onset of depressive symptoms (7, 8). Existing mechanism studies indicate that sleep disorders trigger excessive vigilance, cognitive-emotional dysregulation, and impair prefrontal control functions, which in turn lead to the generation of negative emotions (9). These findings suggest that sleep problems may serve as an early warning sign for broader mental health vulnerabilities among researchers.

Generalized anxiety symptoms represent another critical dimension of mental health status. It is frequently observed as a comorbidity of both insomnia and depression and often precedes the onset of depressive episodes (10). Anxiety symptoms may aggravate sleep disorders by perpetuating hypervigilance and increasing emotional vulnerability, thereby accelerating the transition from insomnia to depression. Existing studies employ the Generalized Anxiety Disorder-7 (GAD-7) scale as a method for screening anxiety symptoms, thus, this study utilizes this scale to assess the level of the anxiety dimension (11). At the same time, psychological resilience has been widely recognized as a protective factor that buffers the influences of stress and sleep disruption (12). It is generally accepted that well resilient individuals often demonstrate more flexible emotion regulation and better recovery from negative experiences, which can mitigate the escalation of distress into depression.

Importantly, longitudinal evidence indicates a reciprocal association between anxiety and resilience (13). In this three-wave cross-lagged study, higher anxiety predicted subsequent reductions in resilience, whereas greater resilience predicted lower later anxiety and depression. Similar studies also indicate that resilience can buffer the mental-health impact of stressors, by moderating the association between pandemic-related impact and anxiety symptoms (14). Occupational studies in medical staff and other frontline workers have reported chain-mediation models, in which pathways of elevated anxiety together with diminished resilience indirectly lead to depression (1, 15). Such findings provide preliminary support for an interactive model of mental health factors, yet this mechanism has rarely been tested among academic researchers, whose psychological profiles may be distinct.

Against this backdrop, we attempt to construct a chained mediation model of mental health levels among Chinese academic researchers, examining how anxiety and psychological resilience mediate the association between insomnia and depressive symptoms. Based on prior evidence, insomnia is expected to show a positive association with depressive symptoms. More critically, we aimed to test an indirect pathway in which higher insomnia is related to heightened anxiety, and resilience further contributes to the link with depression. Furthermore, we examine whether these pathways vary with educational attainment, and recognize that higher educational level may shape distinct psychological processes. By delineating these direct and indirect pathways in academic researchers, the study seeks to clarify how sleep disturbance, anxiety, and resilience interrelate, offering recommendations and references for prevention and intervention methods regarding mental health issues for researchers and research institutions.

## Methods

### Participants and procedure

We conducted an anonymous, cross-sectional online survey of university-affiliated researchers across 19 provinces in China from September 1 to November 21, 2023. This study was conducted in accordance with the ethical principles of the Declaration of Helsinki and its later amendments. The study protocol received approval from the Ethics Committee of the Second Xiangya Hospital. Participants provided electronic informed consent prior to their involvement. Participation was entirely voluntary, and respondents retained the right to withdraw at any time without penalty. Eligible participants were required to hold a full-time or part-time research position at a Chinese university, college, or research institute, encompassing roles such as postdoctoral researchers, faculty, and research staff. Additionally, they were required to have engaged in research-related work during their employment with 18 ≤ age ≤ 65. Individuals who were full-time students without a formal research appointment, international scholars in China primarily for temporary training, or those unable to provide informed consent were excluded from the study. After data collection, we implemented strict quality control procedures. 1) All questions were set as mandatory, and incomplete questionnaires were not collected. 2) A minimum completion time of > 80 seconds was established as a reasonable threshold. 3) For the scale sections, the standard deviation (SD) of responses from each participant was calculated, and questionnaires with an SD of 0 were excluded. Two independent reviewers evaluated whether the questionnaires met the inclusion criteria, and any discrepancies were resolved by a third researcher.

### Self-reported general information

A self-developed form collected demographics and background information, including age, gender, education level (doctoral vs non-doctoral), professional title, research output, and work environment.

### Psychometric scales

For assessment of insomnia symptoms, the Insomnia Severity Index (ISI) (16, 17) was used. The ISI contains 7 items assessing insomnia severity, with total scores ranging from 0 to 28. Scores ≥ 8 indicated screening-defined insomnia. Cronbach’s *α* in this study was 0.925. Additionally, the Generalized Anxiety Disorder 7-item Scale (GAD-7) (18–20) was selected for assessment of anxiety symptoms;This 7-item scale was initially developed to screen for generalized anxiety disorder but has been widely validated as a dimensional measure of general anxiety symptom severity in both clinical and non-clinical populations. Scores from 0 to 21 and higher scores reflect greater anxiety severity. A cutoff of ≥ 5 indicated screening-defined anxiety symptoms. Cronbach’s *α* was 0.932. For assessment of depressive symptoms, the Patient Health Questionnaire 9-item Scale (PHQ-9) (21, 22). The PHQ consists of 9 items evaluating depressive symptoms. Scores ≥ 5 indicated the presence of depression. Cronbach’s *α* was 0.951. Meanwhile, regarding assessment of psychological resilience, the 10-item version of the Connor-Davidson Resilience Scale (CD-RISC) (12, 23, 24) was utilized to measure the level of psychological resilience among participants, and after verification, its Cronbach’s α coefficient in this study was 0.954, achieving an excellent standard of internal consistency reliability.

### Statistical analyses

Analyses were performed in SPSS 26.0; figures were generated in GraphPad Prism 8. Categorical variables were compared using chi-square tests (or Fisher’s exact where cell counts were small). Because scale scores (PHQ, GAD, and ISI) deviated from normality, between-group differences were assessed with Mann–Whitney U tests; results are presented alongside medians and IQRs. Two-sided significance was set at *p*<0.05.

Prior to performing correlation analysis, we conducted Harman’s single-factor test on all scale items as an exploratory screen for common-method variance given the conceptual proximity of anxiety and depression constructs. We then estimated partial correlations among ISI, GAD, PHQ, and CD-RISC, controlling for age and sex. Two-sided *p* values from this correlation matrix were adjusted within-family using the Benjamini–Hochberg false discovery rate (FDR), correlations were considered statistically significant at *p-fdr*<0.05.

### Chain mediation analysis

We estimated a chain mediation model in PROCESS v4.0 (25), specifying ISI as the independent variable, PHQ as the dependent variable, and GAD (M1) and CD-RISC (M2) as mediators; age and gender were included as covariates. We used 5,000 bootstrap resamples to obtain bias-corrected 95% confidence intervals; indirect effects were considered significant when CIs did not include zero. Because the data are cross-sectional, the sequential model is used to test indirect pathways consistent with the proposed ordering; inferences are limited to statistical mediation rather than causation.

### Multi-group structural equation model analysis

To assess potential differences between doctoral and non-doctoral researchers, we conducted a multi-group structural equation model (SEM) in AMOS 26, retaining the same configuration as the chain mediation model for comparability. Structural paths were constrained equal across groups, and χ² difference tests evaluated group differences.

## Results

### Participant characteristics

A total of 821 questionnaires were submitted, of which 645 valid responses were retained after quality control. The proportion meeting the ISI screening threshold (≥ 8) was higher in participants aged ≥ 40 than in those < 40 (64.7% vs 56.4%; χ²(1)=4.57, *p* = 0.032; OR = 1.41, 95%CI[1.03–1.94]) and in doctoral vs non-doctoral researchers (66.0% vs 54.5%; χ²(1)=8.96, *p* = 0.003; OR = 1.62, 95%CI[1.18–2.23]). No significant differences were observed in gender, professional rank, working years, or working roles. Participants who screened positive for insomnia (ISI≥8) showed higher scores on anxiety (GAD), depression (PHQ) and resilience (CD-RISC), compared with non-insomnia counterparts (all *p*<0.001). (**Table 1**; **Supplementary Figure 1**).

**Table 1:** Characteristics of participants with/without insomnia (n = 645).

Variables	Over all (n = 645)	Non-insomnia (n = 256)	Insomnia (n =389)	z/χ2	*p*
Demographic questionnaire					
Gender(%)				1.803	0.194
Male	366(56.7)	137(21.3)	229(35.5)		
Female	279(43.3)	119(18.4)	160(24.8)		
Age(%)				4.572	0.032*
<40years	342(53.0)	149(23.1)	193(29.9)		
≥40years	303(47.0)	107(16.6)	196(30.4)		
Education(%)				8.959	0.003**
PhD-qualified	324(50.2)	110(17.1)	214(33.2)		
Non-PhD-qualified	321(49.8)	146(22.6)	175(27.1)		
Occupational situation					
Professional rank(%)				3.316	0.345
Senior	214(33.2)	76(11.8)	138(21.4)		
Deputy senior	189(29.3)	82(12.7)	107(16.6)		
Intermediate and below	189(29.3)	79(12.2)	110(17.1)		
other	53(8.2)	19(2.9)	34(5.3)		
Working years(%)				4.402	0.111
≤5	255(39.5)	110(17.1)	145(22.5)		
6-10	276(42.8)	110(17.1)	166(25.7)		
>10	114(17.7)	36(5.5)	78(12.1)		
Clinical Position(%)				0.000	0.989
No	350(54.3)	139(21.6)	211(32.7)		
Yes	295(45.7)	117(18.1)	178(27.6)		
Teaching position(%)				0.344	0.557
No	336(52.1)	137(21.2)	199(30.9)		
Yes	309(47.9)	119(18.4)	190(29.5)		
Managerial position(%)				0.312	0.576
No	384(59.5)	149(23.1)	235(36.4)		
Yes	261(40.5)	107(16.6)	154(23.9)		
Psychological questionnaire					
GAD scores	7 (4,16)	4(2,5)	16(8,17)	17.413	<0.001***
CD-RISC scores	25(8,30)	7(5,15.75)	28(23,30)	16.663	<0.001***
PHQ scores	8(5,21)	5(3,6)	20(9,22)	12.514	<0.001***

GAD, Generalized Anxiety Disorder; CD-RISC, Connor-Davidson resilience scale; PHQ, Patient Health Questionnaire. Variables using percentage are reported as a chi-square test between with/without insomnia. All psychological questionnaire data using the Mann-Whitney rank sum test. All data were reported as median (lower, upper quartile). *p<0.05, ** *p* < 0.01, ***p <0.001.

### Correlation analysis

Partial correlations controlling for age and gender revealed that ISI scores were positively associated with GAD, PHQ and CD-RISC scores (all *p-fdr*<0.01). These associations remained consistent across subgroups defined by educational attainment (**Supplementary Table 1**).

### Chain mediation model in the full sample

**Figure 1:**
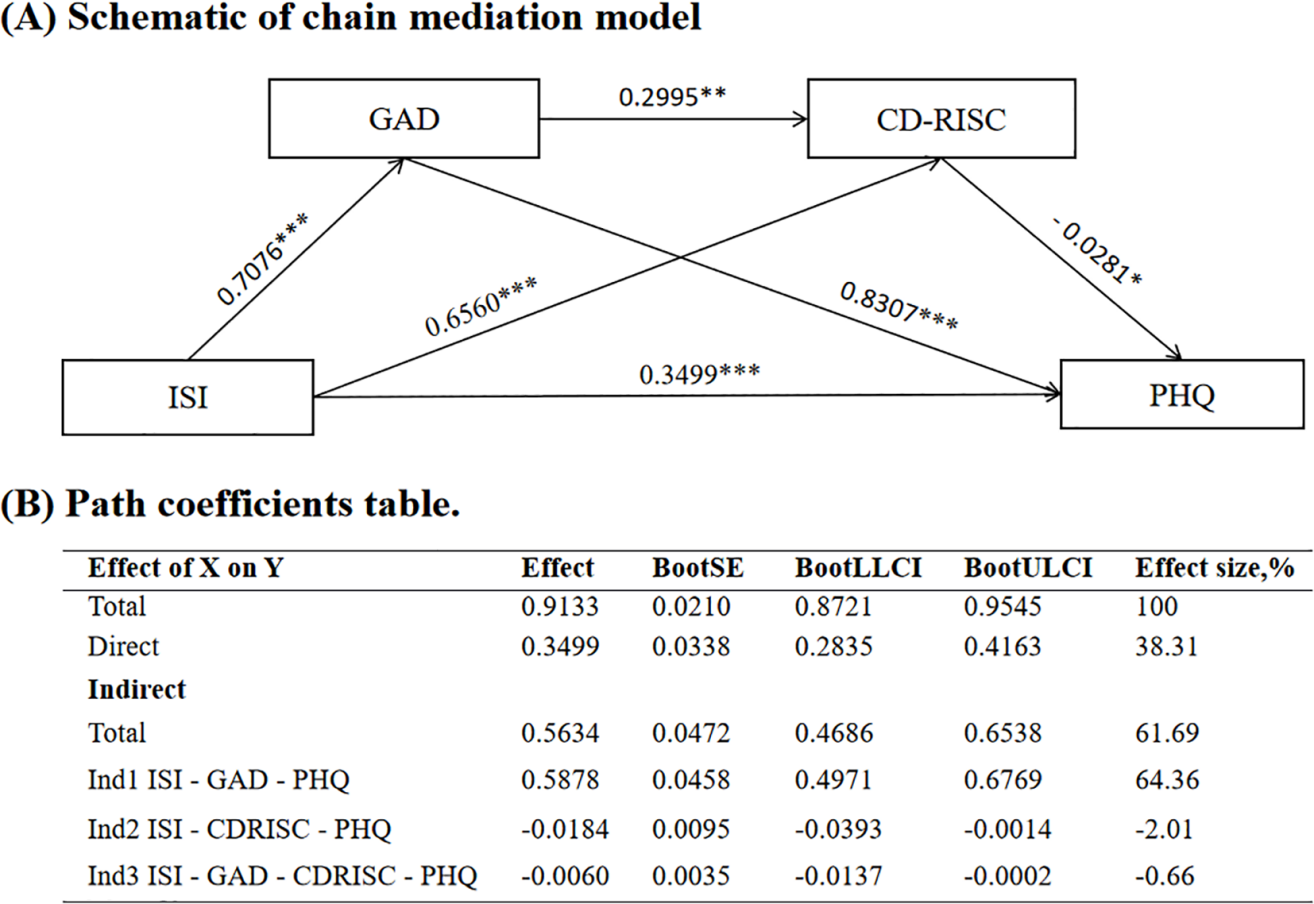
Mediation model of anxiety and resilience between insomnia and depression. **(A)** Schematic of chain mediation model; **(B)** Path coefficients table. ISI, Insomnia Severity Index; GAD, Generalized Anxiety Disorder; CD-RISC, Connor-Davidson resilience scale; PHQ, Patient Health Questionnaire. SE, standard error; LLCI, lower limit of confidence interval; ULCI, upper limit of confidence interval. *p < 0.05, **p < 0.01, ***p < 0.001.

In the chain mediation model, the insomnia had a significant positive effect on anxiety (B = 0.7076, *p*<0.001), resilience (B = 0.6560, *p*<0.001) and depression (B = 0.3499, *p*<0.001). In turn, resilience exerted a significant negative effect on depression (B=–0.0281, *p*<0.05) (**Table 2**, **Figure 1**). The indirect effect was further confirmed using bootstrapping with 5,000 resamples, and the 95% confidence interval excluded zero, indicating robust mediation effects (**Figure 1**).

**Table 2:** Regression analysis of the variable relationship in the mediation model.

Regression equation		Overall fit index			Regression coefficient		
Outcome variables	Predictor variables	R	R2	F	B	t	95%CI
Model 1	Gender	0.8682	0.7537	653.9717	0.6925	2.7652**	(0.2007,1.1843)
(GAD)	Age				-0.0202	-1.1849	(-0.0537,0.0133)
	ISI				0.7076	44.0166***	(0.6760,0.7392)
	GAD				–	–	–
	CD-RISC				–	–	–
Model 2	Gender	0.6065	0.3678	93.0836	-2.8636	-3.953***	(-4.2861,-1.4411)
(CD-RISC)	Age				0.0345	0.7028	(-0.0619,0.1310)
	ISI				0.656	7.0752***	(0.4739,0.8380)
	GAD				0.2995	2.6366**	(0.0764,0.5225)
	CD-RISC				–	–	–
Model 3	Gender	0.9229	0.8518	734.5376	1.0084	3.9146***	(0.5025,1.5142)
(PHQ)	Age				-0.0247	-1.4297	(-0.0586,0.0092)
	ISI				0.3499	10.3457***	(0.2835,0.4163)
	GAD				0.8307	20.7054***	(0.7519,0.9094)
	CD-RISC				-0.0281	-2.0230*	(-0.0554,-0.0008)
Model 4	Gender	0.8674	0.7524	649.1579	1.6582	5.0783***	(1.0170,2.2994)
(PHQ)	Age				-0.0423	-1.9002	(-0.0860,0.0014)
	ISI				0.9133	43.5733***	(0.8721,0.9545)
	GAD				–	–	–
	CD-RISC				–	–	–

ISI, Insomnia Severity Index; GAD, Generalized Anxiety Disorder; CD-RISC, Connor-Davidson resilience scale; PHQ, Patient Health Questionnaire. *, *p* < 0.05, **, *p* < 0.01, ***, *p* < 0.001.

### Multi-group SEM analysis by education

**Figure 2:**
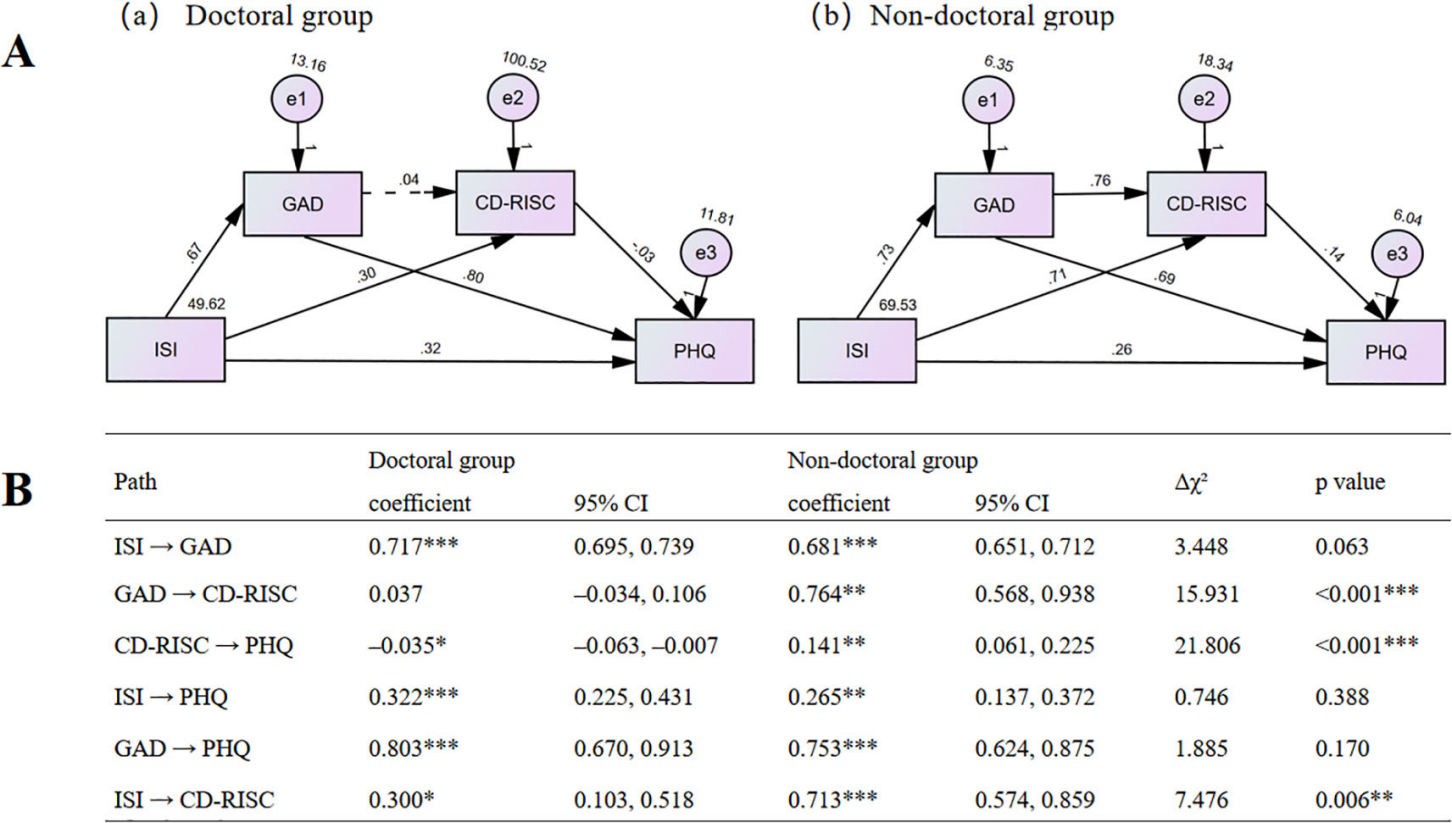
Path diagrams and comparative path coefficients for doctoral and non-doctoral groups. **(A)** Path diagrams of the M-SEM for the doctoral **(a)** and non-doctoral **(b)** groups without path constraints; **(B)** Path Coefficients by Group and Chi-square Difference Tests. Estimates are standardized coefficients. CI = 95% confidence interval. Δχ² is the chi-square difference between the constrained model (each path fixed equal across groups) and the unconstrained model (all paths freely estimated). All chi-square tests used df = 1. *p<0.05, **p < 0.01, ***p <0.001.

Multi-group SEM analysis demonstrated an acceptable fit for both doctoral and non-doctoral groups (**Figure 2**). Among the six hypothesized structural paths, three showed significant group differences when constrained to be equal: GAD → CD-RISC (Δχ²=15.931, *p*<0.001), CD-RISC → PHQ (Δχ²=21.806, *p*<0.001), and ISI → CD-RISC (Δχ²=7.476, *p* = 0.006). Directionally, CD-RISC → PHQ was negative among doctoral researchers but positive among non-doctoral peers (**Figure 2**).

## Discussion

This study examined how insomnia, anxiety, and psychological resilience jointly relate to depressive symptoms in academic researchers, and whether these relations differ by educational level. The research findings are reflected in three aspects. First, insomnia emerged as a central factor of depression, being associated with higher anxiety symptom levels among those who screened positive for insomnia, as well as with indirect statistical associations from sleep disturbance to affective symptoms in the models. Second, anxiety symptoms showed a strong indirect association of the link between insomnia and depression. Third, the role of resilience was more nuanced: while resilience explained a smaller share of indirect effects, its association with depressive symptoms varied by educational level—showing a negative path in researchers with doctoral degree but a positive path in researchers without doctoral degree in the multi-group SEM analysis. Together, these models suggest that insomnia is a central factor of mental health problems in Chinese researchers, whereas the contribution of resilience is educational level dependent.

Insomnia symptoms, as one of the most prevalent mental health issues (26, 27), can prospectively predict the onset of anxiety and depressive symptoms (28). This finding has been supported by multiple longitudinal studies and meta-analyses (29–31). In our model, severe insomnia symptoms co-occurred with higher levels of depression, and insomnia was specified as an upstream correlate; higher insomnia severity was associated with higher anxiety and with depressive symptoms. We speculate that impaired emotion regulation and circadian disruption channel sleep problems into mood dysregulation via anxiety symptoms (32). At the same time, resilience also plays a certain regulatory role in this process.

The mediation models demonstrated that anxiety symptoms accounted for the largest share of the indirect association between insomnia and depressive symptoms. Existing research of insomnia mechanism suggests that, persisting sleep disturbances may exacerbate cognitive–emotional processes intensify, which leading to heightened anxiety symptoms and in turn contribute to depressive burden (30). Bidirectional relationships among sleep disturbance, anxiety, and depression have been found among Chinese cohort studies, and these links are persistent (28, 31). Moreover, evidence from adolescent and young adult populations demonstrates similar sequential patterns: nightmares and sleep disruption predict cognitive deficits and suicidal ideation through combined effects of anxiety and depression (33, 34). Intervention studies provide empirical evidence: RCTs of CBT-I reduce insomnia and are often accompanied by improvements in anxiety and depression, with some studies reporting anxiety reduction as a mediator of mood change (35, 36). Further research suggests that CBT-I alters emotional brain responses by neurocognitive and circadian rhythm interventions, which also support this perspective (37, 38). Collectively, these converging strands position anxiety as a proximal and modifiable correlate of depressive outcomes in high-stress professional contexts.

We also observed a complex role of psychological resilience in the relationship between insomnia and depression among academic professionals. Multigroup structural equation modeling indicated that the moderating effect of resilience on depressive symptoms varied by educational attainment: a negative impact was observed among individuals holding a doctoral degree, whereas a positive impact emerged among those without one. This contrast may stem from differences in occupational resources and autonomy between the two groups. Possessing a doctoral degree is typically associated with greater job security, professional autonomy, and supportive networks (39). These conditions may foster the individuals resilience by promoting recovery and regulating, thereby exerting a protective effect (40). In contrast, non-doctoral researchers often face heightened job insecurity, heavier life burdens, and stronger dependence on supervisors (41, 42). Their resilience may manifest as a passive coping style characterized by endurance and perseverance which can lead to emotional exhaustion and a positive association with depressive symptoms (43, 44).

Cultural values provide a critical contextual framework for understanding how resilience is expressed among Chinese academics. Rooted in Confucian traditions that emphasize diligence, conscientiousness, and collective expectations (45), Chinese researchers often experience substantial normative pressure. Within this cultural milieu, resilience is frequently conceptualized as “endurance” or “persistence”, while emotional suppression is sometimes regarded as an appropriate form of emotion regulation (46). Such cultural expectations shape both doctoral and non-doctoral researchers alike, yet their behavioral consequences diverge due to disparities in access to resources. For those with greater autonomy and occupational security, such as doctoral scholars, perseverance can be transformed into an active and strategic form of resilience that aligns with long-term professional growth (47). Conversely, for non-doctoral researchers with limited resources and fewer alternative coping strategies, the same cultural emphasis may confine their resilience to a passive endurance mode, thereby heightening their vulnerability to psychological distress (48). Hence, culture interacts dynamically with structural factors to jointly shape the mental health outcomes of resilience (49).

Our finding underscores that the psychological benefits of resilience are not universal but are substantially moderated by occupational structure and cultural context. Accordingly, mental health interventions targeting academic populations should adopt a more precise stratified strategies. For resource-limited groups such as non-doctoral and early-career researchers, interventions should prioritize the alleviation of structural stressors, including clarifying job roles, providing transparent career development pathways, and establishing effective supervisory support systems (50–52). Building on this foundation, the implementation of evidence-based approaches such as Cognitive Behavioral Therapy for Insomnia (CBT-I) and Mindfulness-Based Stress Reduction (MBSR) may help individuals transition from passive endurance to adaptive self-regulation (53). Ultimately, institutional efforts toward equitable resource allocation and the institutionalization of a supportive research culture represent the fundamental path to ensuring the sustainable psychological well-being of the entire scientific ecosystem (54).

This study has several limitations. First, the design is cross-sectional; temporal ordering cannot be determined, and causal inferences require longitudinal and intervention designs. We specified an *a priori* ordering and estimated indirect associations with bias-corrected bootstraps, but longitudinal follow-ups would clarify temporal dynamics. Second, all measures were based on self-report scales, which may introduce common-method bias and underreporting of psychological distress due to social desirability or stigma surrounding mental health in academic settings. The absence of diagnostic screening for past or current psychiatric disorders also limits the ability to distinguish subclinical symptoms from clinical conditions. Future studies could combine self-report data with objective sleep indices, clinical assessments, and diagnostic screening to improve measurement precision and validity. Finally, we focused on the core pathway and did not include several work-environment variables (e.g., workload patterns, shift schedules) that could further refine effect estimates; future studies can add these to enhance the practical significance of research.

## Conclusion

In conclusion, our findings support a model in which insomnia and anxiety form the principal pathway to depressive symptoms among researchers, whereas the influence of resilience varies across educational and occupational contexts. Interventions that target sleep health, reduce anxiety, and address modifiable features of the work environment—alongside calibrated resilience-building—are likely to deliver the most reliable mental-health benefit in academic settings.

## Data Availability

The raw data supporting the conclusions of this article will be made available by the authors, without undue reservation.
