# Supplementary material for: Insomnia and depression in Chinese academic researchers: mediation by anxiety and resilience with differences among researchers by educational level
Source: Front Psychiatry. 2025 Nov 21;16:1709399. doi: 10.3389/fpsyt.2025.1709399 (PMC12679156; doi:10.3389/fpsyt.2025.1709399)
Supplement: Supplementary file 1 [file SupplementaryFile1.docx]

## **Supplementary figure 1.**

### Differences in psychometric scales between insomnia and non-insomnia researchers. Note. (A) Difference in GAD (Generalized Anxiety Disorder) total scores between insomnia and no insomnia group; (B) Difference in total CD-RISC (Connor-Davidson resilience scale) scores between insomnia and no-insomnia groups; (C) Differences in PHQ (Patient Health Questionnaire) scores between the insomnia and non-insomnia groups. Mann-Whitney U test, ****p* < 0.001.


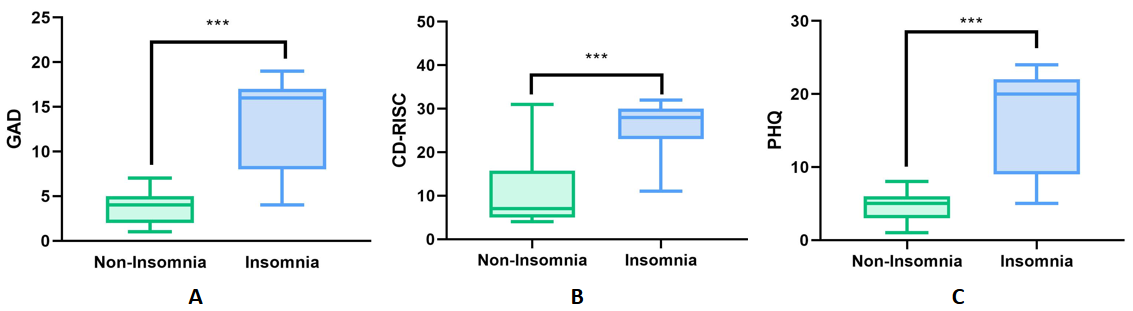


## Supplementary table 1.

**Robust partial correlations among insomnia, anxiety, depression, and resilience in the full sample and by educational subgroup.** Note. ISI, Insomnia Severity Index; GAD, Generalized Anxiety Disorder; CD-RISC, Connor-Davidson resilience scale; PHQ, Patient Health Questionnaire. ***p-fdr* <0.01, ****p-fdr*  <0.001.

| **Subject** | **Variables** | **ISI** | **GAD** | **PHQ** | **CD-RISC** |
| --- | --- | --- | --- | --- | --- |
| Total sample  (N=645) | ISI | 1 |  |  |  |
|  | GAD | 0.867*** | 1 |  |  |
|  | PHQ | 0.594*** | 0.556*** | 1 |  |
|  | CD-RISC | 0.865*** | 0.907*** | 0.515*** | 1 |
| Doctoral group  (N=324) | ISI | 1 |  |  |  |
|  | GAD | 0.796*** | 1 |  |  |
|  | PHQ | 0.806*** | 0.869*** | 1 |  |
|  | CD-RISC | 0.225*** | 0.195*** | 0.155** | 1 |
| Non-doctoral group  (N=321) | ISI | 1 |  |  |  |
|  | GAD | 0.924*** | 1 |  |  |
|  | PHQ | 0.925*** | 0.945*** | 1 |  |
|  | CD-RISC | 0.914*** | 0.908*** | 0.911*** | 1 |
|  | | | | | |
